# A Mathematical Realization of Entropy through Neutron Slowing Down

**DOI:** 10.3390/e20040233

**Published:** 2018-03-28

**Authors:** Barry Ganapol, Domiziano Mostacci, Vincenzo Molinari

**Affiliations:** 1 Department of Aerospace and Mechanical engineering, University of Arizona, Tucson, AZ 85721, USA; 2 Department of Industrial Engineering, University of Bologna, 40136 Bologna, Italy

**Keywords:** entropy, elastic scattering, neutron slowing down

## Abstract

The slowing down equation for elastic scattering of neutrons in an infinite homogeneous medium is solved analytically by decomposing the neutron energy spectrum into collision intervals. Since scattering physically smooths energy distributions by redistributing neutron energy uniformly, it is informative to observe how mathematics accommodates the scattering process, which increases entropy through disorder.

## 1. Introduction

Neutron slowing down in an infinite homogeneous medium [1] is a classic problem in neutron transport theory. Neutrons (test particles) collide elastically with nuclei (field particles) and thereby lose energy to nuclear recoil. Thus, we have a common collisional process as described by a balance in energy phase space between a neutron source and neutrons scattering into and out of an infinitesimal energy increment, leading to the slowing down equation. One can analytically solve this equation for the neutron collision density distribution as it tends toward its equilibrium state. In addition, neutron loss is possible through radiative capture but will not be considered. Limiting our investigation to an infinite medium has naturally eliminated spatial and directional variation. While the slowing down equation is deterministic, it nevertheless describes the statistical scattering process, as illustrated by the associated mathematics.

In the following, we argue that the solution to the neutron slowing down equation characterizes the evolution of disorder associated with neutron–nucleus collisions. While it is not strictly correct to attribute disorder to entropy [2], in our case, starting from monoenergetic neutrons representing complete order, subsequent scattering creates disorder by uniformly redistributing neutron energy and recoil energy transfer to field particles. The nucleus scattering model conserves kinetic energy; however, it should be noted that the slowing down process assumes background nuclei are at rest. This considerably simplifies the scattering kernel and allows an analytical solution. Beginning with oscillations of the collision density in lethargy (logarithm of energy), called Placzek transients, neutron slowing down demonstrates increasing entropy with increasing lethargy. The oscillations originate from the discontinuity of derivatives submerged further into the solution at collision interval boundaries. As will be shown, the initially sharp discontinuity from the singular delta function source embeds itself in higher-order derivatives. Hence, with increasing lethargy, the solution becomes smoother, which is a tendency toward increased randomness and equilibrium. Therefore, neutron slowing down is a physical example of the mathematical representation of increasing disorder since one begins with a source of zero entropy (certainty), and, with an ever-increasing number of collision intervals, smoothing (uncertainty) of the energy distribution follows.

## 2. Solution

### 2.1. The Slowing Down Equation

The neutron slowing down equation in a purely scatter material in the fast neutron regime is

(1a) $$F\left(E\right)=\int_{0}^{E_{0}^{+}}dE^{\prime }P\left(E^{\prime }\to E\right)F\left(E^{\prime }\right)+\delta \left(E-E_{0}\right)$$

for the collision (energy) density

(1b) $$F\left(E\right)\equiv \Sigma_{s}\left(E\right)\phi \left(E\right),$$

where $\Sigma_{s}\left(E\right)$ is the scattering cross section and ϕ(E) is the neutron scalar flux. The neutron scatters elastically from a nucleus uniformly to the energy interval $\alpha E^{\prime }\leq E\leq E^{\prime }$ with probability of scattering into dE given by

(1c) $$P\left(E^{\prime }\to E\right)dE=\frac{dE}{\left(1-\alpha \right)E^{\prime }};$$

otherwise, the probability is zero. The scattering parameter is

(1d) $$\alpha \equiv {\left(\frac{A-1}{A+1}\right)}^{2},$$

where *A* is the mass number of the nucleus, and a monoenergetic source emits neutrons at energy $E_{0}$. Therefore, Equation (1a) becomes

(2) $$F\left(E\right)=\frac{1}{1-\alpha }\int_{E}^{\min\left(E_{0}^{+},E/\alpha \right)}\frac{dE^{\prime }}{E^{\prime }}F\left(E^{\prime }\right)+\delta \left(E-E_{0}\right).$$

Note that to include source neutrons, the upper limit in the scattering integral must come from just above $E_{0}$.

#### Change to Lethargy Variable

With the change of energy to the lethargy variable,

$$u\equiv \ln\left(\frac{E_{0}}{E}\right),$$

Equation (2) becomes

(3a) $$F\left(u\right)=\frac{1}{1-\alpha }\int_{\max\left(0^{-},\;u-q\right)}^{u}due^{-\left(u-u^{\prime }\right)}F\left(u^{\prime }\right)+\delta \left(u\right),$$

where

(3b) $$q\equiv \ln\left(\frac{1}{\alpha }\right);$$

and with the further transformation

(4a) $$F\left(u\right)\equiv e^{-u}g\left(u\right),$$

there results,

(4b) $$g\left(u\right)=\frac{1}{1-\alpha }\int_{\max\left(0^{-},u-q\right)}^{u}du^{\prime }g\left(u^{\prime }\right)+\delta \left(u\right).$$

### 2.2. Determination of g(u) by Collision Interval

**Figure 1:**
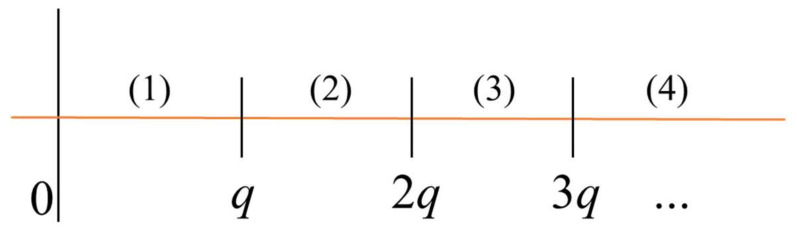
Scattering collision intervals (*n* = 1, 2, 3, 4).

A natural decomposition of lethargy into scattering collision intervals (*n*), shown in Figure 1, enables an explicit solution. The lethargy interval *q* is the maximum lethargy gain a neutron experiences after a single collision.

#### 2.2.1. Collision Interval (1)

In the first collision interval (1), Equation (4b) is

(5a) $$g_{1}\left(u\right)=\frac{1}{1-\alpha }\int_{0}^{u}du^{\prime }g_{1}\left(u^{\prime }\right)+\delta \left(u\right).$$

For

(5b) $$g\left(u\right)\equiv g_{1}\left(u\right),\;0\leq u\leq q,$$

a convenient solution is

(6a) $$g_{1}\left(u\right)=g_{0}\left(u\right)+g_{1c}\left(u\right).$$

The source, emitting uncollided neutrons, defines

(6b) $$g_{0}\left(u\right)\equiv \delta \left(u\right),$$

and introducing Equation (6a) into Equation (5a) gives

(6c) $$g_{1c}\left(u\right)=\frac{1}{1-\alpha }\int_{0}^{u}du^{\prime }g_{1c}\left(u^{\prime }\right)+\frac{1}{1-\alpha }$$

for neutrons experiencing at least one collision. Therefore, upon differentiation

(7a) $$\frac{dg_{1c}\left(u\right)}{du}=\frac{1}{1-\alpha }g_{1c}\left(u\right),$$

and solving, with initial condition

(7b) $$g_{1c}\left(0\right)=\frac{1}{1-\alpha },$$

from Equation (6c)

(7c) $$g_{1c}\left(u\right)=\frac{1}{1-\alpha }e^{u/\left(1-\alpha \right)}.$$

The solution in interval (1) is

(8) $$g_{1}\left(u\right)=\delta \left(u\right)+\frac{1}{1-\alpha }e^{u/\left(1-\alpha \right)},$$

exhibiting the delta function source discontinuity at *u* = 0 with no disorder and source neutrons scattering to the end of interval (1).

#### 2.2.2. Collision Interval (2)

For the second collision interval, q≤u≤2q, Equation (4b) becomes

$$g\left(u\right)=\frac{1}{1-\alpha }\int_{u-q}^{u}du^{\prime }g\left(u^{\prime }\right);$$

and, if

(9a) $$g\left(u\right)\equiv g_{2}\left(u\right),\;q\leq u\leq 2q,$$

then

(9b) $$g_{2}\left(u\right)=\frac{1}{1-\alpha }\int_{u-q}^{q}du^{\prime }g_{1}\left(u^{\prime }\right)+\frac{1}{1-\alpha }\int_{q}^{u}du^{\prime }g_{2}\left(u^{\prime }\right),$$

where scattering from interval (1) contributes to interval (2). Differentiating gives

(9c) $$\frac{dg_{2}\left(u\right)}{du}=\frac{1}{1-\alpha }g_{2}\left(u\right)-\frac{1}{{\left(1-\alpha \right)}^{2}}e^{\left(u-q\right)/\left(1-\alpha \right)}-\frac{1}{1-\alpha }\delta \left(u-q\right).$$

Before solving Equation (9c), we note the delta function source singularity, originally at *u* = 0, has moved to the derivative of $g_{2}\left(u\right)$ at *u* = *q*, and continues on to higher derivatives in subsequent collision intervals, as will be shown.

From Equation (9b),

(9d) $$g_{2}\left(q^{+}\right)=\underset{\varepsilon \to 0}{\lim}g_{2}\left(q+\varepsilon \right)=\frac{1}{1-\alpha }\int_{0^{+}}^{q}du^{\prime }g_{1}\left(u^{\prime }\right)=\frac{1}{1-\alpha }\left[e^{q/\left(1-\alpha \right)}-1\right];$$

and on solving Equation (9c) as a sum of the solution to the homogeneous equation and the particular solution gives

(9e) $$g_{2}\left(u\right)=\left[g_{2}\left(q^{+}\right)-\frac{\left(u-q\right)}{{\left(1-\alpha \right)}^{2}}\right]e^{\left(u-q\right)/\left(1-\alpha \right)}+\frac{1}{1-\alpha }\left[1-\Theta \left(u-q\right)\right],$$

or, since from Equation (8),

$$g_{1}\left(0^{+}\right)=\frac{1}{1-\alpha },$$

(9f) $$g_{2}\left(u\right)=\left[g_{2}\left(q^{+}\right)-\frac{\left(u-q\right)}{\left(1-\alpha \right)}g_{1}\left(0^{+}\right)\right]e^{\left(u-q\right)/\left(1-\alpha \right)}+\frac{1}{1-\alpha }\left[1-\Theta \left(u-q\right)\right].$$

Though the last term vanishes in interval (2), it is theoretically necessary to give the delta function discontinuity in the derivative.

Note that since Equation (8) evaluated at $q^{-}$ is

(10a) $$g_{1}\left(q^{-}\right)=\frac{1}{1-\alpha }e^{q/\left(1-\alpha \right)},$$

across the boundary of intervals (1) and (2), one observes a finite discontinuity in g(u),

(10b) $$\Delta g_{2}\left(q\right)\equiv g_{2}\left(q^{+}\right)-g_{1}\left(q^{-}\right)=-\frac{1}{1-\alpha };$$

hence, the delta function in Equation (9c) at *u* = *q* in the derivative of $g_{2}\left(u\right)$.

#### 2.2.3. Collision Interval (3)

To establish a pattern, we continue to interval (3) with Equation (4b) for *n* = 3:

(11a) $$g_{3}\left(u\right)=\frac{1}{1-\alpha }\int_{u-q}^{2q}du^{\prime }g_{2}\left(u^{\prime }\right)+\frac{1}{1-\alpha }\int_{2q}^{u}du^{\prime }g_{3}\left(u^{\prime }\right),$$

where

(11b) $$g\left(u\right)\equiv g_{3}\left(u\right),\;2q\leq u\leq 3q.$$

On differentiation of Equation (11a):

(11c) $$\frac{dg_{3}\left(u\right)}{du}=\frac{1}{1-\alpha }g_{3}\left(u\right)-\frac{1}{1-\alpha }g_{2}\left(u-q\right)$$

and solving

$$g_{3}\left(u\right)=g_{3}\left(2q^{+}\right)e^{\left(u-2q\right)/\left(1-\alpha \right)}-\frac{1}{1-\alpha }\int_{2q}^{u}du^{\prime }e^{\left(u-u^{\prime }\right)/\left(1-\alpha \right)}g_{2}\left(u^{\prime }-q\right).$$

After integration of the last term, we find

(11d) $$g_{3}\left(u\right)=\left[g_{3}\left(2q^{+}\right)-\frac{\left(u-2q\right)}{\left(1-\alpha \right)}g_{2}\left(q^{+}\right)+\frac{1}{2}\frac{{\left(u-2q\right)}^{2}}{{\left(1-\alpha \right)}^{3}}\right]e^{\left(u-2q\right)/\left(1-\alpha \right)},$$

which is also

(11e) $$g_{3}\left(u\right)=\left[g_{3}\left(2q^{+}\right)-\frac{\left(u-2q\right)}{\left(1-\alpha \right)}g_{2}\left(q^{+}\right)+\frac{1}{2}\frac{{\left(u-2q\right)}^{2}}{{\left(1-\alpha \right)}^{2}}g_{1}\left(0^{+}\right)\right]e^{\left(u-2q\right)/\left(1-\alpha \right)}.$$

The initial condition, $g_{3}\left(2q^{+}\right)$, for interval (3) is approached from within the interval and is given by Equation (11a) as

$$g_{3}\left(2q^{+}\right)=\underset{\varepsilon \to 0}{\lim}g_{3}\left(2q+\varepsilon \right)=\frac{1}{1-\alpha }\int_{q}^{2q}du^{\prime }g_{2}\left(u^{\prime }\right).$$

However, from Equation (9b), we also find

(11f) $$g_{2}\left(2q^{-}\right)=\frac{1}{1-\alpha }\int_{q}^{2q}du^{\prime }g_{2}\left(u^{\prime }\right),$$

demonstrating the continuity of g(2q) across intervals (2) and (3), and completing the solution for collision interval (3).

#### 2.2.4. Collision Interval (*n*)

##### Continuity for $u>2q^{+}$

In general, defining the solution $g_{n}\left(u\right)$ for interval (*n*),

(12a) $$g\left(u\right)=g_{n}\left(u\right),\;\left(n-1\right)q\leq u\leq nq,$$

and partitioning the scattering integral in Equation (4b) into current and previous intervals gives

(12b) $$g_{n}\left(u\right)=\frac{1}{1-\alpha }\int_{u-q}^{\left(n-1\right)q}du^{\prime }g_{n-1}\left(u^{\prime }\right)+\frac{1}{1-\alpha }\int_{\left(n-1\right)q}^{u}du^{\prime }g_{n}\left(u^{\prime }\right).$$

**Figure 2:**
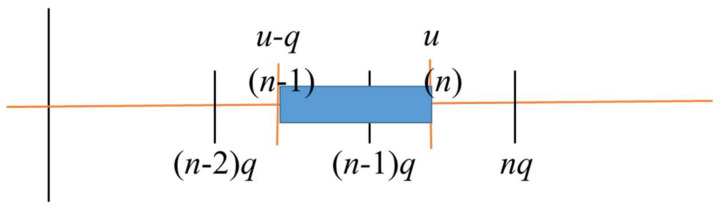
Contribution from previous collision interval-.

The interpretation of this expression is shown in Figure 2, and, as already mentioned, includes a contribution to interval (*n*) from scatter in the previous interval.

For n≥3, from Equation (12b) with *n* decremented by unity and *u* = $\left(n-1\right)q-\varepsilon \to \left(n-1\right)q^{-}$, in the limit as ε→0:

(13a) $$g_{n-1}\left(\left(n-1\right)q^{-}\right)=\underset{\varepsilon \to 0}{\lim}g_{n-1}\left(\left(n-1\right)q-\varepsilon \right)=\frac{1}{1-\alpha }\int_{\left(n-2\right)q}^{\left(n-1\right)q}du^{\prime }g_{n-1}\left(u^{\prime }\right).$$

Similarly, for *u* = $\left(n-1\right)q+\varepsilon \to \left(n-1\right)q^{+}$ in Equation (12b),

(13b) $$g_{n}\left(\left(n-1\right)q^{+}\right)=\underset{\varepsilon \to 0}{\lim}g_{n}\left(\left(n-1\right)q+\varepsilon \right)=\frac{1}{1-\alpha }\int_{\left(n-2\right)q}^{\left(n-1\right)q}du^{\prime }g_{n-1}\left(u^{\prime }\right)$$

and therefore, comparing to Equation (13a),

(13c) $$\Delta g_{n}\left(\left(n-1\right)q\right)=g_{n}\left(\left(n-1\right)q^{+}\right)-g_{n-1}\left(\left(n-1\right)q^{-}\right)=0.$$

Thus, g(u) is a continuous function with the exception of the delta function singularity at *u* = 0 and the finite discontinuity at u=q.

#### 2.2.5. General Solution

From $g_{n}\left(u\right),n=2,3$, the following pattern emerges:

(14) $$g_{n}\left(u\right)=e^{\left(u-\left(n-1\right)q\right)/\left(1-\alpha \right)}\sum_{k=0}^{n-1}\gamma_{n,k}{\left(u-\left(n-1\right)q\right)}^{k}+\frac{1}{1-\alpha }\left[1-\Theta \left(u-q\right)\right]\delta_{n,2},$$

where for *n* = 2

$$\begin{array}{l} \gamma_{2,0}=g_{2}\left(q^{+}\right)\\ \gamma_{2,1}=-\frac{1}{\left(1-\alpha \right)}g_{1}\left(0^{+}\right), \end{array}$$

and *n* = 3

$$\begin{array}{l} \gamma_{3,0}=g_{3}\left(2q\right)\\ \gamma_{3,1}=-\frac{1}{\left(1-\alpha \right)}g_{2}\left(q^{+}\right)\\ \gamma_{3,2}=\frac{1}{2}\frac{1}{{\left(1-\alpha \right)}^{2}}g_{1}\left(0^{+}\right). \end{array}$$

We now confirm the pattern by constructive inductive reasoning.

For n≥4, assume the form of the solution, Equation (14), is true for *n* − 1

(15a) $$g_{n-1}\left(u\right)=e^{\left(u-\left(n-2\right)q\right)/\left(1-\alpha \right)}\sum_{k=0}^{n-2}\gamma_{n-1,k}{\left(u-\left(n-2\right)q\right)}^{k}.$$

Differentiating Equation (12b) gives the ODE:

(15b) $$\frac{dg_{n}\left(u\right)}{du}=\frac{1}{1-\alpha }g_{n}\left(u\right)-\frac{1}{1-\alpha }g_{n-1}\left(u-q\right),$$

with the following solution over the interval $(\left(n-1\right)q^{+}$, $nq^{-})$:

(15c) $$\begin{array}{ll} g_{n}\left(u\right) & =g_{n}\left(\left(n-1\right)q^{+}\right)e^{\left(u-\left(n-1\right)q\right)/\left(1-\alpha \right)}\\ & -\frac{1}{1-\alpha }\int_{\left(n-1\right)q^{+}}^{u}du^{\prime }e^{\left(u-u^{\prime }\right)/\left(1-\alpha \right)}e^{\left(u^{\prime }-\left(n-1\right)q\right)/\left(1-\alpha \right)}\sum_{k=0}^{n-2}\gamma_{n-1,k}{\left(u^{\prime }-\left(n-1\right)q\right)}^{k}. \end{array}$$

Performing the integration:

(15d) $$g_{n}\left(u\right)=\left[g_{n}\left(\left(n-1\right)q^{+}\right)-\frac{1}{1-\alpha }\sum_{k=0}^{n-2}\frac{\gamma_{n-1,k}}{k+1}{\left(u-\left(n-1\right)q\right)}^{k+1}\right]e^{\left(u-\left(n-1\right)q\right)/\left(1-\alpha \right)},$$

and decrementing index *k* by unity gives

(15e) $$g_{n}\left(u\right)=\left[g_{n}\left(\left(n-1\right)q^{+}\right)-\frac{1}{1-\alpha }\sum_{k=1}^{n-1}\frac{\gamma_{n-1,k-1}}{k}{\left(u-\left(n-1\right)q\right)}^{k}\right]e^{\left(u-\left(n-1\right)q\right)/\left(1-\alpha \right)}.$$

Hence, on comparison to Equation (14), for *k* = 0:

(16a) $$\gamma_{n,0}=g_{n}\left(\left(n-1\right)q^{+}\right),$$

and for  k=1,2,…

(16b) $$\gamma_{n,k}=-\frac{1}{\left(1-\alpha \right)k}\gamma_{n-1,k-1}$$

On solving the recurrence of Equations (16):

(16c) $$\gamma_{n,k}=\frac{{\left(-1\right)}^{k}}{{\left(1-\alpha \right)}^{k}k!}\gamma_{n-k,0}=\frac{{\left(-1\right)}^{k}}{{\left(1-\alpha \right)}^{k}k!}g_{n-k}\left(\left(n-\left(k+1\right)\right)q^{+}\right),$$

we have the conjectured solution of the form of Equation (14) for intervals n≥3:

(16d) $$g_{n}\left(u\right)=e^{\left(u-\left(n-1\right)q\right)/\left(1-\alpha \right)}\sum_{k=0}^{n-1}\frac{{\left(-1\right)}^{k}}{k!}{\left[\frac{u-\left(n-1\right)q}{1-\alpha }\right]}^{k}g_{n-k}\left(\left(n-\left(k+1\right)\right)q^{+}\right).$$

Or, from the continuity of g(u) (Equation (13c)),

(17a) $$g_{n}\left(u\right)=e^{\left(u-\left(n-1\right)q\right)/\left(1-\alpha \right)}\left[\begin{array}{l} g_{n-1}\left(\left(n-1\right)\tilde{q}\right)\\ +\sum_{k=1}^{n-1}\frac{{\left(-1\right)}^{k}}{k!}{\left[\frac{u-\left(n-1\right)q}{1-\alpha }\right]}^{k}g_{n-k}\left(\left(n-\left(k+1\right)\right)\tilde{q}\right) \end{array}\right],$$

where

(17b) $$\left(n-\left(k+1\right)\right)\tilde{q}=\{\begin{array}{l} \;0^{+},\;k=n-1\\ 2q^{+},\;k=n-2\\ \left(n-\left(k+1\right)\right)q,\;1\leq k\leq n-3. \end{array}$$

From Equation (4a), the collision density by collision interval is therefore

(18a) $$F_{n}\left(u\right)=e^{\left(\alpha u-\left(n-1\right)q\right)/\left(1-\alpha \right)}\left[\begin{array}{l} g_{n-1}\left(\left(n-1\right)\tilde{q}\right)\\ +\sum_{k=1}^{n-1}\frac{{\left(-1\right)}^{k}}{k!}{\left[\frac{u-\left(n-1\right)q}{1-\alpha }\right]}^{k}g_{n-k}\left(\left(n-\left(k+1\right)\right)\tilde{q}\right) \end{array}\right],$$

For future use, the *j*th derivative (using Leibnitz’s rule) is:

(18b) $$\begin{array}{l} F_{n}^{\left(j\right)}\left(u\right)\\ =e^{\left(\alpha u-\left(n-1\right)q\right)/\left(1-\alpha \right)}\left[\begin{array}{r} g_{n-1}\left(\left(n-1\right)\tilde{q}\right)\delta_{j0}+\sum_{k=1}^{n-1}\frac{{\left(-1\right)}^{k}}{k!}{\left[\frac{1}{1-\alpha }\right]}^{k}g_{n-k}\left(\left(n-\left(k+1\right)\right)\tilde{q}\right)\\ ∙\sum_{l=0}^{j}\frac{j!}{l!\left(j-l\right)!}{\left[\frac{\alpha }{1-\alpha }\right]}^{j-l}{\left[u-\left(n-1\right)q\right]}^{k-l} \end{array}\right]. \end{array}$$

We now investigate the singularities of derivatives of g(u).

## 3. Continuity/Singularities

So far, we have identified the singularities given in Table 1. In this section, all the relevant singularities starting at interval (*n*) will also be identified. To do so, we require several conjectures concerning the continuity of the collision density.

**Conjecture** **1.** *The jth derivatives of* $g_{n-1}$ *and* $g_{n}$ *at u = (n − 1)q for* n≥j+3 *are continuous.*

Symbolically, Conjecture 1 is

(C1.1) $$\Delta g_{n}^{\left(j\right)}\left(\left(n-1\right)q\right)\equiv g_{n}^{\left(j\right)}\left(\left(n-1\right)q^{+}\right)-g_{n-1}^{\left(j\right)}\left(\left(n-1\right)q^{-}\right)=0,\;n\geq j+3,$$

which has already been shown for *j* = 0 above (Equation (13c)).

Assuming the conjecture true for *j* − 1 gives

(C1.2) $$\Delta g_{n}^{\left(j-1\right)}\left(\left(n-1\right)q\right)=g_{n}^{\left(j-1\right)}\left(\left(n-1\right)q^{+}\right)-g_{n-1}^{\left(j-1\right)}\left(\left(n-1\right)q^{-}\right)=0,\;n\geq j+2.$$

We next apply *j* − 1 derivatives to the differentiation of Equation (15b) to give

(C1.3) $$g_{n}^{\left(j\right)}\left(u\right)=\frac{1}{1-\alpha }\left[g_{n}^{\left(j-1\right)}\left(u\right)-g_{n-1}^{\left(j-1\right)}\left(u-q\right)\right];$$

moreover, if we reduce *n* by unity, then

(C1.4) $$g_{n-1}^{\left(j\right)}\left(u\right)=\frac{1}{1-\alpha }\left[g_{n-1}^{\left(j-1\right)}\left(u\right)-g_{n-2}^{\left(j-1\right)}\left(u-q\right)\right].$$

Evaluating Equations (C1.3) and (C1.4) at (*n* − 1)*q*^+^ and (*n* − 1)*q*^−^, respectively,

(C1.5) $$g_{n}^{\left(j\right)}\left(\left(n-1\right)q^{+}\right)=\frac{1}{1-\alpha }\left[g_{n}^{\left(j-1\right)}\left(\left(n-1\right)q^{+}\right)-g_{n-1}^{\left(j-1\right)}\left(\left(n-2\right)q^{+}\right)\right]$$

(C1.6) $$g_{n-1}^{\left(j\right)}\left(\left(n-1\right)q^{-}\right)=\frac{1}{1-\alpha }\left[g_{n-1}^{\left(j-1\right)}\left(\left(n-1\right)q^{-}\right)-g_{n-2}^{\left(j-1\right)}\left(\left(n-2\right)q^{-}\right)\right]$$

and subtracting

(C1.7) $$\Delta g_{n}^{\left(j\right)}\left(\left(n-1\right)q\right)=\frac{1}{1-\alpha }\left\{\begin{array}{l} \left[g_{n}^{\left(j-1\right)}\left(\left(n-1\right)q^{+}\right)-g_{n-1}^{\left(j-1\right)}\left(\left(n-2\right)q^{+}\right)\right]-\\ -\left[g_{n-1}^{\left(j-1\right)}\left(\left(n-1\right)q^{-}\right)-g_{n-2}^{\left(j-1\right)}\left(\left(n-2\right)q^{-}\right)\right] \end{array}\right\}.$$

On re-arrangement,

(C1.8) $$\Delta g_{n}^{\left(j\right)}\left(\left(n-1\right)q\right)=\frac{1}{1-\alpha }\left\{\begin{array}{l} \left[g_{n}^{\left(j-1\right)}\left(\left(n-1\right)q^{+}\right)-g_{n-1}^{\left(j-1\right)}\left(\left(n-1\right)q^{-}\right)\right]-\\ -\left[g_{n-1}^{\left(j-1\right)}\left(\left(n-2\right)q^{+}\right)-g_{n-2}^{\left(j-1\right)}\left(\left(n-2\right)q^{-}\right)\right] \end{array}\right\},$$

which is

(C1.9) $$\Delta g_{n}^{\left(j\right)}\left(\left(n-1\right)q\right)=\frac{1}{1-\alpha }\left\{\Delta g_{n}^{\left(j-1\right)}\left(\left(n-1\right)q\right)-\Delta g_{n-1}^{\left(j-1\right)}\left(\left(n-2\right)q\right)\right\}.$$

Since, by assumption, the first term is

(C1.10) $$\Delta g_{n}^{\left(j-1\right)}\left(\left(n-1\right)q\right)=0,\;n\geq j+2;$$

and with *n* replaced by *n* − 1, the second term is

(C1.11) $$\Delta g_{n-1}^{\left(j-1\right)}\left(\left(n-2\right)q\right)=0,\;n\geq j+3.$$

Thus, Equation (C1.9) vanishes and confirms Conjecture 1, which is therefore true by induction.

**Conjecture** **2.** *The n* − *2 derivative at u = (n* − *1)q is discontinuous for* $g_{n},n\geq 2.$

Symbolically, Conjecture 2 is

(C2.1) $$\Delta g_{n}^{\left(n-2\right)}\left(\left(n-1\right)q\right)\neq 0,\;n\geq 2.$$

We have already shown for *n* = 2:

(C2.2) $$\Delta g_{2}^{\left(0\right)}\left(q\right)=g_{2}\left(q^{+}\right)-g_{1}\left(q^{-}\right)=-\frac{1}{1-\alpha }\neq 0.$$

Assume conjecture is true for *n*−1:

(C2.3) $$\Delta g_{n-1}^{\left(n-3\right)}\left(\left(n-2)\right)q\right)\neq 0,\;n\geq 3.$$

Introduce *j* = *n* − 2 into Equation (C1.9):

(C2.4) $$\Delta g_{n}^{\left(n-2\right)}\left(\left(n-1\right)q\right)=\frac{1}{1-\alpha }\left\{\Delta g_{n}^{\left(n-3\right)}\left(\left(n-1\right)q\right)-\Delta g_{n-1}^{\left(n-3\right)}\left(\left(n-2\right)q\right)\right\}.$$

However, from Conjecture 1, Equation (C1.1):

(C2.5) $$\Delta g_{n}^{\left(j\right)}\left(\left(n-1\right)q\right)=0,\;n\geq j+3,$$

which implies for j=n−3≥0

(C2.6) $$\Delta g_{n}^{\left(n-3\right)}\left(\left(n-1\right)q\right)=0;$$

and Equation (C2.4) by assumption becomes

(C2.7) $$\Delta g_{n}^{\left(n-2\right)}\left(\left(n-1\right)q\right)=-\Delta g_{n-1}^{\left(n-3\right)}\left(\left(n-2\right)q\right)\neq 0,$$

which is Conjecture 2—again, proved by induction.

Solving the recurrence in Equation (C2.7) gives the discontinuity

(C2.8) $$\Delta g_{n}^{\left(n-2\right)}\left(\left(n-1\right)q\right)=\frac{{\left(-1\right)}^{n-1}}{{\left(1-\alpha \right)}^{n-1}}.$$

**Conjecture** **3.** *The n − 1 derivative of* $g_{n}$ *at u = (n − 1)q contains a delta function singularity.*

Symbolically, Conjecture 3 is

(C3.1) $$g_{n}^{\left(n-1\right)}\left(u\right)=h_{n}\left(u\right)+\beta_{n}\delta \left(u-\left(n-1\right)q\right),\;n\geq 1.$$

We have shown that Conjecture 3 is true for *n* = 1 (and *n* = 2)

(C3.2) $$g_{1}^{\left(0\right)}\left(u\right)=h_{1}\left(u\right)+\beta_{1}\delta \left(u\right)$$

in Equation (8) with

(C3.3) $$\begin{array}{l} \beta_{1}\left(u\right)=1\\ h_{1}=\frac{1}{1-\alpha }e^{u/\left(1-\alpha \right)}. \end{array}$$

Assuming the conjecture is true for *n* − 1:

(C3.4) $$g_{n-1}^{\left(n-2\right)}\left(u\right)=h_{n-1}\left(u\right)+\beta_{n-1}\delta \left(u-\left(n-2\right)q\right),\;n\geq 2.$$

From Equation (C1.2) with *j* = *n* − 1:

(C3.5) $$g_{n}^{\left(n-1\right)}\left(u\right)=\frac{1}{1-\alpha }\left[g_{n}^{\left(n-2\right)}\left(u\right)-g_{n-1}^{\left(n-2\right)}\left(u-q\right)\right]$$

and Equation (C3.4) becomes

(C3.6) $$\begin{array}{ll} g_{n}^{\left(n-1\right)}\left(u\right) & =\frac{1}{1-\alpha }\left[g_{n}^{\left(n-2\right)}\left(u\right)-h_{n-1}\left(u-q\right)\right]-\frac{\beta_{n-1}}{1-\alpha }\delta \left(u-q-\left(n-2\right)q\right)\\ & =h_{n}\left(u\right)+\beta_{n}\delta \left(u-\left(n-1\right)q\right), \end{array}$$

where

(C3.7) $$\begin{array}{l} h_{n}\left(u\right)\equiv \frac{1}{1-\alpha }\left[g_{n}^{\left(n-2\right)}\left(u\right)-h_{n-1}\left(u-q\right)\right]\\ \beta_{n}\equiv -\frac{\beta_{n-1}}{1-\alpha }, \end{array}$$

which is Conjecture 3. In addition,

(C3.8) $$\beta_{n}\equiv \frac{{\left(-1\right)}^{n-1}}{{\left(1-\alpha \right)}^{n-1}}.$$

In summary, from the three conjectures and the analytical solution of Equation (17), one concludes

(a) $g_{n}\left(u\right)$ is continuous at *u* = (*n* − 1)*q* for n≥3 and within the interval
  (n−1)q≤u≤nq;
(b) $g_{n}^{\left(n-2\right)}\left(u\right)$ has a finite discontinuity at *u* = (*n* − 1)*q* for n≥2 and is otherwise continuous;
(c) $g_{n}^{\left(n-1\right)}\left(u\right)$ has a delta function discontinuity at *u* = (*n* − 1)*q* for n≥2 and is otherwise continuous.

Finally, the derivatives of collision density *F*(*u*) inherit the continuity properties of *g*(*u*), since, by Leibnitz’s rule,

(19) $$\begin{array}{ll} F_{n}^{\left(j\right)}\left(u\right) & =\frac{d^{j}}{du^{j}}\left[e^{-u}g_{n}\left(u\right)\right]\\ & =e^{-u}\sum_{l=0}^{j}{\left(-1\right)}^{l-j}\frac{j!}{l!\left(l-j\right)!}g_{n}^{\left(l\right)}\left(u\right);\;\left(n-1\right),\;q\leq u\leq nq. \end{array}$$

## 4. Singularities and Smoothing

**Figure 3:**
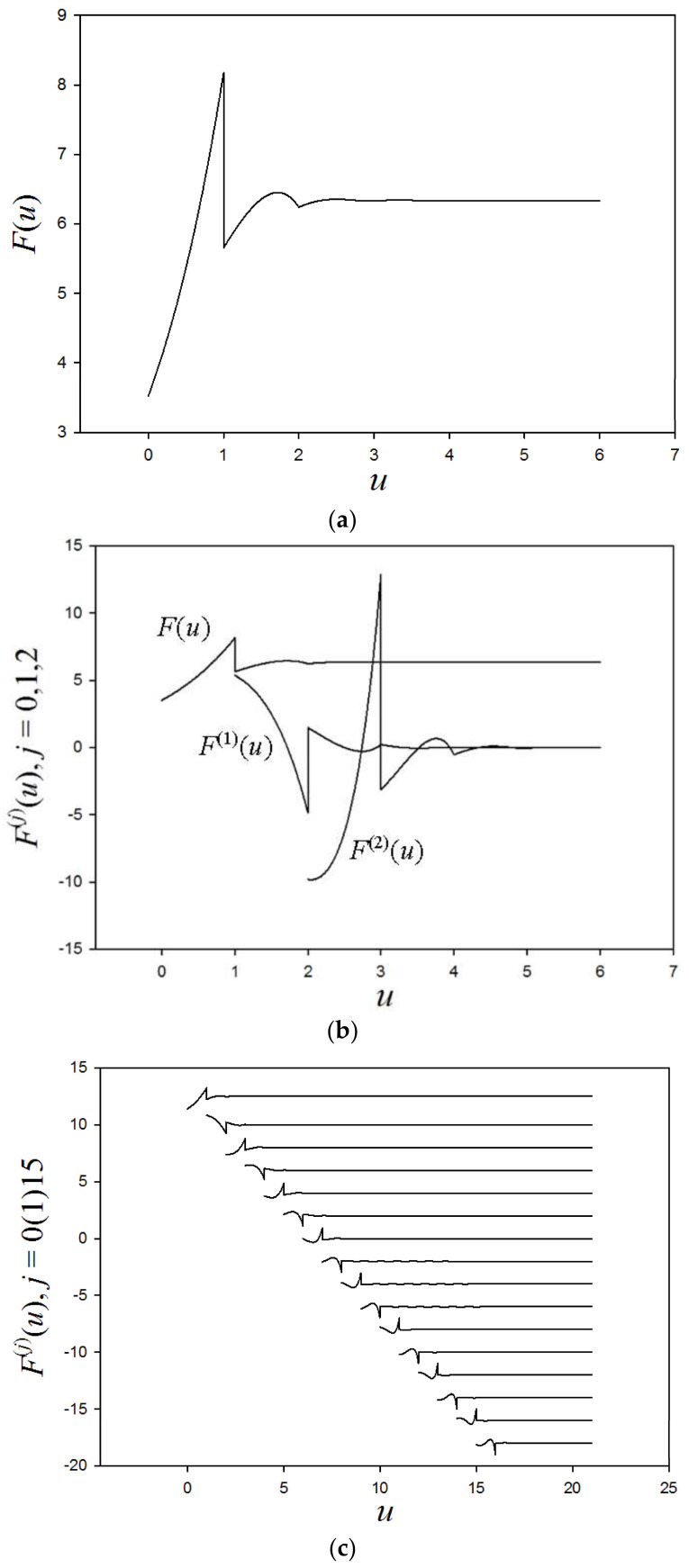
(**a**) Collision density; (**b**) first two derivative of collision density and the collision density; (**c**) 15 derivatives of collision density.

Table 2, displaying points of discontinuity of the collision density for scattering against ^12^C, is based on the above continuity arguments. To the right is increasing lethargy and down the rows increasing derivatives. As is apparent, with increasing lethargy (and disorder), discontinuities become further embedded in the collision density derivatives, making *F*(*u*) ever smoother. Embedding of the discontinuities is clearly observed in Figure 3a–c. As noted above, the finite discontinuity at *u* = *q*, from integration over the delta function source emerges in the collision density itself, as shown in Figure 3a.

As neutrons scatter to lower energy (higher lethargy), the memory of the singular source is retained since a delta function (not shown) exists at the beginning of each scattering interval exactly where the previous derivative has a finite discontinuity.

Figure 3b shows several derivatives, as given by Equation (18b). We observe Placzek oscillations in *F*(*u*), including the finite discontinuity at *u* = *q*. The oscillations for increasing *u* obviously originate from the submerged discontinuities and are indicative of increasing entropy (disorder) and smoothing as the influence of the discontinuities becomes further submerged in the derivatives. Also observed is the constant asymptotic collision density (1/*E*) equilibrium distribution, as shown in Table 3, where

$$F\left(\infty \right)=1/\left[\alpha +\frac{\alpha }{1-\alpha }\ln\left(\alpha \right)\right].$$

Figure 3c shows 15 derivatives normalized so that the finite discontinuity in each is the same, and the derivatives are displaced downward for better viewing. The pattern is evident and visualizes how increasing physical smoothing is mathematically linked to the submergence of the initial discontinuities of the source distribution with lethargy.

## 5. Randomness of Collisions

Another statistical measure of the disorder in the collision density distribution is the randomness of collisions. In the following analysis, we find an expression for the distribution of the collided density in terms of the number of collisions.

As shown in [3], the Laplace transform of Equation (3a) gives the following transform of the collision density:

(20a) $$\bar{F}\left(p\right)=\frac{1}{1-Q_{s}\left(p\right)},$$

with

(20b) $$Q_{s}\left(p\right)=\frac{c}{1-\alpha }\left[\frac{1-e^{-q\left(p+1\right)}}{p+1}\right].$$

Here, *c* is the number of neutrons emitted in a scattering collision relative to the total possible interactions including loss by absorption. In the analysis above, *c* is unity.

The geometric series representation of Equation (20a) is

(21) $$\bar{F}\left(p\right)=\sum_{n=0}^{\infty }Q_{s}{\left(p\right)}^{n}=\sum_{n=0}^{\infty }\frac{c^{n}}{{\left(1-\alpha \right)}^{n}}{\left[\frac{1-e^{-q\left(p+1\right)}}{p+1}\right]}^{n},$$

where convergence is guaranteed by choice of the complex variable *p*. Then, using the binomial theorem for the term in brackets, Equation (21) becomes

(22a) $$\bar{F}\left(p\right)=\sum_{n=0}^{\infty }\frac{c^{n}}{{\left(1-\alpha \right)}^{n}}\sum_{l=0}^{n}{\left(-1\right)}^{l}\frac{n!}{\left(n-l\right)!l!}\left[\frac{e^{-ql\left(p+1\right)}}{{\left(p+1\right)}^{n}}\right],$$

whose analytical inversion becomes

(22b) $$F\left(u\right)=\delta \left(u\right)+\sum_{n=1}^{\infty }c^{n}\left[e^{-u}\sum_{l=0}^{\left[u/q\right]}{\left(-1\right)}^{l}\frac{n!}{\left(n-l\right)!l!}{\left(\frac{u-lq}{1-\alpha }\right)}^{n-1}\right].$$

The upper limit of the second summation [u/q] is the greatest integer contained in *u*/*q*. Note that *n* is now the collision number, not to be confused with the collision interval.

The first term in Equation (22b) is the uncollided collision density at the source and the term in brackets is the *n*th collided collision density after *n* collision:

(23a) $$F_{0}\left(u\right)=\delta \left(u\right)$$

(23b) $$F_{n}\left(u\right)\equiv \frac{e^{-u}}{(n-1)!}\sum_{l=0}^{\left[u/q\right]}{\left(-1\right)}^{l}\frac{n!}{\left(n-l\right)!l!}{\left(\frac{u-lq}{1-\alpha }\right)}^{n-1};\;n=1,2,\ldots ,$$

where the subscript is the number of collisions and *c* is unity for purely scattering.

**Figure 4:**
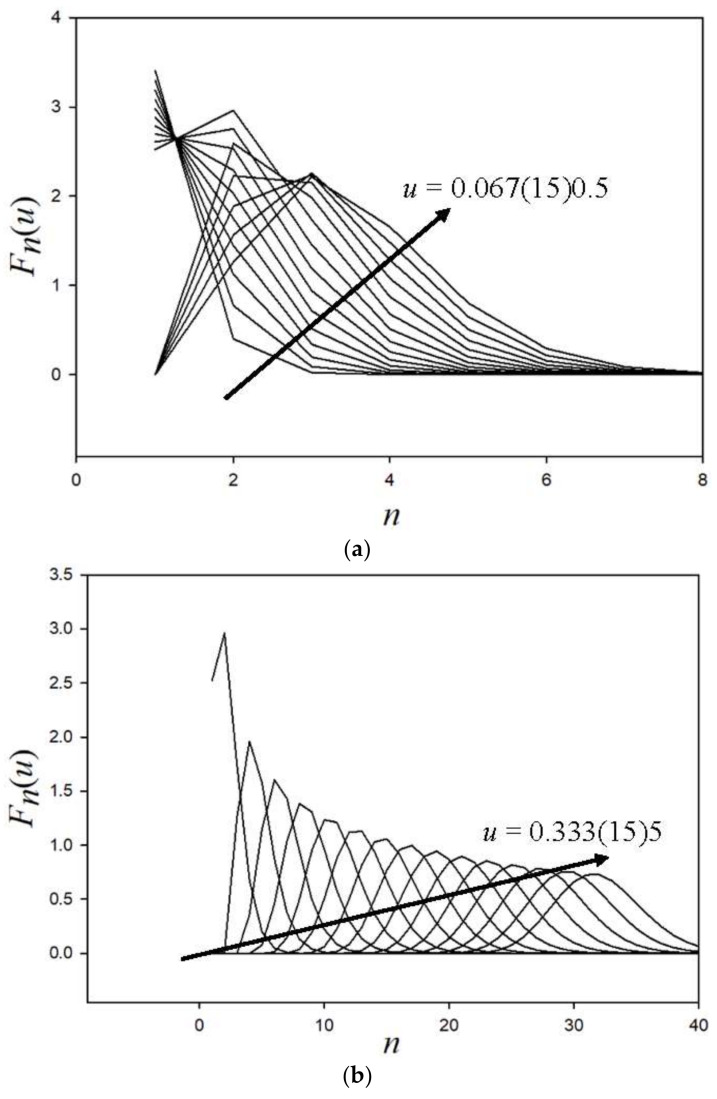
(**a**) Collided collision density to *u* = 0.5; (**b**) Collided collision density to *u* = 5.

For lethargy less than 0.5, Figure 4a shows the variation of the collided density with *n*, where the hint of a Gaussian distribution is observed. As lethargy increases, as anticipated, the development of the Gaussian distribution becomes evident in Figure 4b, verifying the randomness of the neutron–nucleus collision. The numerical evaluation of Equation (23b) is highly sensitive to round-off error and requires quadruple precision, which increases the computing time (normally under a minute) on a LENOVO 2.4 GHz YOGA platform by several seconds.

How do we know that the distribution is indeed Gaussian? This can be shown relatively easily by noting that a normalized Gaussian frequency is

(24a) $$f\left(x\right)=\frac{1}{\sqrt{2\pi \sigma^{2}}}e^{{\left(x-\mu \right)}^{2}/2\sigma^{2}}.$$

If each curve of Figure 4b is a Gaussian, then when, at each lethargy *u*, the distribution is normalized by the area under the curve, which is F(u),

(24b) $$\hat{f}\left(x\right)=\frac{F_{x}\left(u\right)}{\sum_{n=0}^{\infty }F_{n}\left(u\right)},$$

where *x* is now a continuous analogue of the collision number *n*. By equating Equations (24a) and (24b) at their maxima *μ*:

(24c) $$\frac{1}{\sqrt{2\pi \sigma^{2}}}=\hat{f}\left(\mu \left(u\right)\right),$$

where the collision number at maximum is

(24d) $$\mu \left(u\right)=\frac{\sum_{n=0}^{\infty }nF_{n}\left(u\right)}{\sum_{n=0}^{\infty }F_{n}\left(u\right)},$$

there results

(24e) $$\sigma {\left(u\right)}^{2}=\frac{1}{2\pi \hat{f}{\left(\mu \left(u\right)\right)}^{2}}.$$

**Figure 5:**
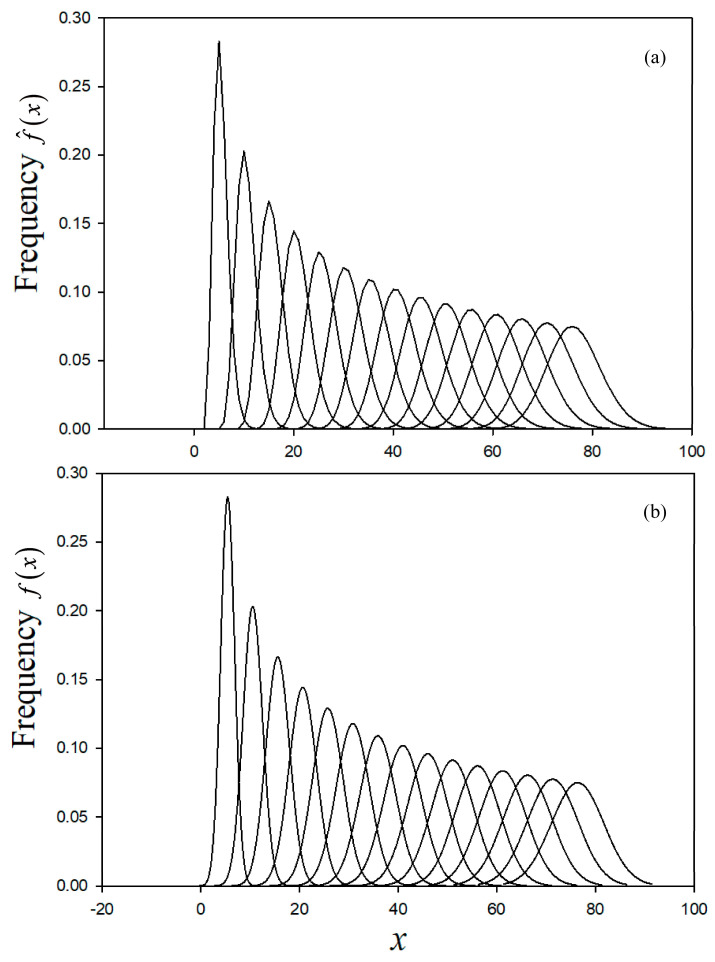
Comparison of calculated frequency of collision (**a**) and the corresponding Gaussian (**b**).

With the parameters for the Gaussian now known, we can plot the two distributions as shown in Figure 5. They are nearly graphically identical over *u* = [0.8, 12].

## 6. Conclusions

Through a rather involved, rigorous mathematical derivation, verification of the obvious was achieved. In particular, the connection between the increased physical smoothness of the collision density distribution with lethargy and consequent singularities from monoenergetic source emission was demonstrated. It was shown that, with increased collisions, the original source singularity becomes submerged in the derivatives of the distribution function, resulting in smoothing of the distribution function. In addition, the tendency of the collision frequency over the number of collisions to become Gaussian with increased lethargy was also demonstrated.

## Figures and Tables

**Table 1:** Singularities identified.

Interval	*u*	Derivative (*j*)	Type
1	0	0	Infinite *
2	*q*	0	Finite
2	*q*	1	Infinite *

* Delta function.

**Table 2:** Embedding of discontinuities in *j*th derivative of *F*(*u*) with collision interval *n*.

(*n* − 1)/(*n*) *j*/*u*	0 0	(1)/(2) *q*	(2)/(3) 2*q*	(3)/(4) 3*q*	(4)/(5) 4*q*	(5)/(6) 5*q*	… …
0	DF	F	C	C	C	C	…
1	-	DF	F	C	C	C	…
2	-	-	DF	F	C	C	…
3	-	-	-	DF	F	C	…
4	-	-	-	-	DF	F	…
5	-	-	-	-	-	DF	…
…	-	-	-	-	-	-	…

C: Continuous; F: Finite discontinuity; DF: Delta function.

**Table 3:** Run-up to asymptotic collision density.

*u*	*F*(*u*)
6	6.3383867546
8	6.3383811228
10	6.3383812067
12	6.3383812061
∞	6.3383812061
